# Identification of Altered miRNAs in Cerumen of Dogs Affected by Otitis Externa

**DOI:** 10.3389/fimmu.2020.00914

**Published:** 2020-05-29

**Authors:** Cristina Lecchi, Valentina Zamarian, Giorgia Borriello, Giorgio Galiero, Guido Grilli, Mario Caniatti, Elisa Silvia D'Urso, Paola Roccabianca, Roberta Perego, Michela Minero, Sara Legnani, Raffaele Calogero, Maddalena Arigoni, Fabrizio Ceciliani

**Affiliations:** ^1^Dipartimento di Medicina Veterinaria, Università degli Studi di Milano, Milan, Italy; ^2^Dipartimento di Sanità Animale, Istituto Zooprofilattico Sperimentale del Mezzogiorno, Portici, Italy; ^3^Department of Small Animal Clinical Science, Institute of Veterinary Science, University of Liverpool, Liverpool, United Kingdom; ^4^Department of Biotechnology and Health Sciences, Molecular Biotechnology Center, Università di Torino, Turin, Italy

**Keywords:** otitis externa, dogs, miRNA, sequencing, biomarkers

## Abstract

Otitis externa is one of the most common diseases in dogs. It is associated with bacteria and yeast, which are regarded as secondary causes. Cerumen is a biological substance playing an important role in the protection of ear skin. The involvement of cerumen in immune defense is poorly understood. MicroRNAs can modulate the host immune response and can provide promising biomarkers for several inflammatory and infectious disorder diagnosis. The aims of this study were to profile the cerumen miRNA signature associated with otitis externa in dogs, integrate miRNAs to their target genes related to immune functions, and investigate their potential use as biomarkers. Cerumen was collected from healthy and otitis affected dogs and the expression of miRNAs was profiled by Next Generation Sequencing; the validation of the altered miRNAs was performed using RT-qPCR. The potential ability of miRNAs to modulate immune-related genes was investigated using bioinformatics tools. The results pointed out that 32 miRNAs, of which 14 were up- and 18 down-regulated, were differentially expressed in healthy vs. otitis-affected dogs. These results were verified by RT-qPCR. To assess the diagnostic value of miRNAs, ROC analysis was carried out, highlighting that 4 miRNAs are potential biomarkers to discriminate otitis-affected dogs. Bioinformatics showed that cerumen miRNAs may be involved in the modulation of host immune response. In conclusion, we have demonstrated for the first time that miRNAs can be efficiently extracted and quantified from cerumen, that their profile changes between healthy and otitis affected dogs, and that they may serve as potential biomarkers. Further studies are necessary to confirm their diagnostic value and to investigate their interaction with immune-related genes.

## Introduction

Otitis externa is defined as the inflammation of the external ear canal and represents one of the most prevalent skin disorders in dogs (1–4). The causes of otitis externa can be divided into primary and secondary (5, 6). Primary causes of otitis include inflammatory conditions, such as autoimmune or immune-mediated diseases, keratinization and glandular disorders, and ectoparasites. Onset of secondary causes such as bacterial and *Malassezia* spp. infections is generally associated by the emergence of primary diseases such as canine atopic dermatitis or combined with several predisposing factors (7). The host immune response to microorganisms in the external ear canal likely plays a pivotal role, but few data are available in dogs, except for studies on the immune reaction against *Malassezia* (8–10). Cerumen, or earwax, is a biological substance composed of lipids, proteins, amino acids, and carbohydrates produced by the combination of the excretions of ceruminous and sebaceous glands in the auditory canal of the external ear of mammalians. Cerumen is believed to protect the epithelial lining of the ear canal against pathogens. Besides its importance as a physical barrier, the involvement of cerumen in other functions, including specific immune defense, remains largely unexplored. Cerumen is supposedly involved in antimicrobial defense as demonstrated by the presence of lysozyme and immunoglobulins (11), as well as of additional proteins with antimicrobial functions, as recently shown by proteomics (12). In human cerumen, proteins belonging to β-defensin families were also detected (13), suggesting a possible role in the local innate immune response.

In addition to its biological function, cerumen has gained interest in the clinical setting as a potential source of biomarkers (14). Cerumen composition indeed reflects the pathophysiological status of the patient, containing lipids, proteins, and metabolites derived from blood (14). Although the amount and the variation of texture and color of cerumen during ear diseases have been accurately described in dogs (15), the active protective role of cerumen in the development of immunity during otitis externa is yet to be determined.

MicroRNAs (miRNAs) are short (~22 nucleotides), single-stranded non-coding RNAs that modulate gene expression by binding to complementary target mRNA. MiRNAs down-regulate gene expression by silencing or degrading their mRNA target (16). Extensive research over the last years demonstrated that miRNAs fulfill a fundamental role in pathogen recognition and inflammatory responses (17). The profile of miRNAs is tissue-dependent and relative stable during several disorders and pathological alterations. Therefore, besides their importance as regulators of immune defenses and inflammation, miRNAs also provide promising targets and biomarkers for molecular-based diagnostics and therapies in both humans (18) and animals (19–21). Changes in miRNAs expression pattern have been observed in association with skin diseases (22) and in otitis media, where they were located in middle ear fluid exosomes (23, 24).

Since no information on miRNAs derangements in canine otitis externa is available, and that cerumen might provide a source of biomarkers, the present study aimed to assess miRNAs expression profiles in the cerumen of dogs affected by otitis externa. This study tested the hypothesis that (a) cerumen microRNA could be differentially abundant between healthy and otitis affected dogs; (b) cerumen microRNA could provide a source of biomarkers to discriminate between healthy and otitis-affected dogs, and (c) cerumen could be a source of microRNAs involved in immune reaction, and as such participates to the regulation of ear innate immunity. A next-generation sequencing pilot study was carried out to identify a list of potential differentially expressed (DE)-miRNAs extracted from cerumen. Results were validated and quantified by RT-qPCR, and functional enrichment analysis of target genes and functional interaction network analysis was finally carried out to identify pathways potentially affected by DE-miRNAs.

## Materials and Methods

### Subjects and Sample Collection

The study was prospective, randomized, and blinded. Twenty client-owned dogs, of which 16 with bilateral and 4 with unilateral bacterial otitis externa, were included. Written informed consent was secured from dog owners prior to enrolment. Diagnosis of otitis externa was based on history, clinical signs such as head shaking, pruritus, local pain, otorrhea, erythema, or swelling of at least one ear canal, visible debris and discharge in the ear canal upon otoscopic examination, and cytological confirmation of bacterial overgrowth and/or bacterial infection by microscopic examination of the exudate. To collect the ear exudate, the external ear canal of the right and left ear was swabbed and the non-sterile cotton-tipped swabs obtained were streaked onto two glass slides, which were then heat-fixed and stained with a modified Wright's stain (Quick Panoptic Kit; Pokler Italia). At least 10 fields per slide were examined under optical microscopy and a number of bacteria ≥25 per high power microscopy field (400×), with or without bacterial phagocytosis by neutrophil granulocytes, were considered positive (infection) as previously described (25). Dogs with any topical or ongoing treatments for otitis externa were excluded. In the control group, 28 dogs deemed healthy based on history, physical, and otoscopic examination and on the absence of neutrophil granulocytes, bacteria <25 and yeast <5 per high power microscopical field (400x) on ear cytology (25) were included. Supplementary Table 1 summarizes the characteristics of dogs.

After inclusion, the skin of each vertical ear canal was sampled by rubbing (I) a tubed sterile dry swab™ rayon [ref MW1028; MWE Co (Bath) LTD—England] for small RNA extraction; (II) a Transystem AMIES w/o charcoal plastic applicator rayon tipped swab (Copan Italia SPA—Brescia—Italy) for microbiological test; and (III) a non-sterile cotton-tipped swab for cytology for 10 s.

### Microbiological Analysis

Microbiological analyses were performed as previously reported (26). Each swab was plated on Blood Agar plates with 5% sheep blood (Thermo Fisher Scientific), Mannitol Salt Agar (Thermo Fisher Scientific), and Mac Conkey agar (Thermo Fisher Scientific); the plates were aerobically incubated at 37°C for 24–48 h.

The same samples were plated on Sabouraud's dextrose agar with chloramphenicol and incubated at 30°C for 7 days and were used to identify the fungal flora.

The isolated bacteria were identified according to standard laboratory procedures (morphology, Gram staining, catalase, oxidase, etc.) and subjected to biochemical identification using the API system (bioMèerieux SA, Marcy L'Etoile, France). The species identification by miniaturized biochemical tests was accepted when the probability was >90%.

### Cytology

The ear swabs were air-dried and stained with May-Grünwald Giemsa. The following parameters were evaluated at the microscope: cellularity, presence of epithelial cells, inflammatory cells, bacteria, and *Malassezia* spp. A semi-quantitative scoring system to evaluate all the parameters was designed. A five-point scale scoring was proposed as follows: 0, absent; 1, very rare presence; 2, mild presence (scarce number in some microscopic fields); 3, moderate presence (variable number in almost all microscopic fields); or 4, good presence (good number in all microscopic fields). When bacteria were present, their morphology was recorded.

### Small RNA Extraction and Sequencing

Total RNA was extracted using miRNeasy Serum/Plasma Kit (Qiagen, Cat. No 217184) following the manufacturer's instruction. The RNA quality and quantity were verified according to MIQE guidelines (27). For all samples, RNA concentration was quantified by Qubit^®^ 2.0 Fluorometer with ^Qubit®^ microRNA Assay Kit (Invitrogen, Cat. No. Q32880).

A pilot sequencing was performed on 3 healthy (Supplementary Table 1, no. 15 right, 19 right, and 20 right) and 3 otitis-affected samples (Supplementary Table 1, no. 32 left, 35 left, and 41 right). Small RNA transcripts were converted into barcoded cDNA libraries. Library preparation was performed as previously reported (28) using the NEBNext Multiplex small RNA Library Prep Set (Cat. No. NEB#E7560) for Illumina and run on the NextSeq500 (Illumina Inc., USA).

### Computational Analyses

The output of NextSeq500 Illumina sequencer was demultiplexed using bcl2fastq Illumina software embedded in docker4seq package (29). miRNA expression quantification was performed using the workflow previously described (30), using the implementation as previously described (31). In brief, after adapter trimming with cutadapt (32), sequences were mapped using SHRIMP (33) to *Canis familiaris* precursors miRNAs available in miRBase 22.0—March 2018 (http://www.mirbase.org/). Counts table and cpm tables were used.

### Validation by RT-qPCR

Total RNA was extracted from all samples included in the study using miRNeasy Serum/Plasma Kit (Qiagen, Cat. No. 217184). One ml of Qiazol (Qiagen) was added and, after incubation at room temperature for 5 min, 3.75 μl (25 fmol final concentration) of the exogenous synthetic spike-in control *Caenorhabditis elegans* miRNA cel-miR-39 (Qiagen, Cat. No. 219610) was spiked into samples. The reverse transcription was performed using the TaqMan Advanced miRNA cDNA Synthesis Kit (Applied Biosystems, Cat. No. A28007) as per manufacturer's instruction. The qPCR experiments were designed following MIQE guidelines (27). The small RNA TaqMan assays were performed according to the manufacturer's instructions using the selected primer/probe assays (ThermoFisher Scientific), including: cel-miR-39-3p (assay ID 478293_mir); cfa-miR-21-5p (assay ID rno481342_mir); cfa-miR-26a-5p (assay ID mmu481013_mir); cfa-miR-27b-3p (assay ID rno478270_mir); cfa-miR-320a-3p (assay ID 478594_mir); cfa-miR-342-3p (assay ID 478043_mir); cfa-miR-146a-5p (assay ID 478399_mir); cfa-miR-378a-3p (assay ID 478349_mir); cfa-miR-375-3p (assay ID mmu481141_mir); cfa-miR-423-5p (assay ID mmu481834_mir); miR-125b (assay ID rno480907_mir); and miR-199 (custom-designed). miRNAs were selected among those with the highest read counts. Quantitative reactions were performed in duplicate in scaled down (15 μl) reaction volumes using the TaqMan Fast Advanced Master Mix (Applied Biosystems, Cat. No. 4444558) on CFX96 Real-Time PCR detection system (BioRad Laboratories). The standard cycling program was 50°C for 2 min, 95°C for 3 min, and 40 cycles of 95°C for 10 s and 60°C for 30 s. Endogenous control for qPCR normalization was identified adapting the pipeline developed by Eisenberg and Levanon (34). Briefly, reference miRNAs were selected considering the individual raw count and with at least 50 reads for each sample; a standard error of the log_2_ fold change value <0.75 and a log_2_ fold change ranging between −0.074 and 0.46. Three reference miRNAs (cfa-miR-21, cfa-miR-26a, and cfa-miR27b) have been selected. No-RT controls and no-template controls were performed. The geometric mean of reference miRNA abundance was used for normalization. The relative quantification of target miRNAs was carried out after normalization of the sample using the geometric mean of reference miRNAs.

### miRNA Target Prioritization

The target genes of DE-miRNAs were predicted using MiRWalk 3.0 (35), which includes 3 miRNA-target prediction programs [miRDB (36), miRTarBase (37), and Targetscan (38)]. The analysis was performed targeting the entire gene sequence (including 5′UTR, CDS, and 3′UTR). The list of target genes predicted by the three tools was included in further analysis and functional mRNA enrichment was performed using DAVID (Database for Annotation, Visualization and Integrated Discovery) bioinformatic resource (39, 40) and biological pathways in the KEGG (Kyoto Encyclopedia of Genes and Genomes) (41) were examined for enrichment. To visualize the interaction between immune-related genes and up- and down-regulated miRNAs, miRNet (42) software was employed to construct the miRNA-hub gene networks.

### Computational and Statistical Analysis

Raw reads quality-check, adapter clipping, and mapping were performed as previously reported (30). After reads mapping, a matrix of integer values was created. The value in the *i*-th row and the *j*-th column of the matrix reported how many reads have been unambiguously assigned to mature miRNA *i* in the sample *j*. The unwanted variation present in the data was estimated using the functions implemented in the SVA package (43). The differential expression analysis was run using DESeq2 (44), setting a thresholds adjusted *p* < 0.1 and |log_2_FC| >1. The differential expressed miRNAs (DE-miRNAs) were those associated with adjusted False Discovery Rate (FDR) ≤ 0.05 and the mean read count ≥300.

Statistical analysis was carried out using XLStat for Windows (Addinsoft, New York, U.S.A.), IBM SPSS Statistics 25 software (IBM Corp., 2017) and MedCalc 14.0 (MedCalc Software bvba, Ostend, Belgium). Statistical significance was accepted at *p* < 0.05. Data were tested for normality and homogeneity of variance using the Kolmogorov-Smirnov and Levene tests, respectively. As data were not normally distributed, non-parametric statistical tests were applied. The Kruskal-Wallis test was used to assess differences in miRNAs concentrations. *P*-values were adjusted using the Bonferroni correction.

A multivariate statistical analysis (Principal Component Analysis—PCA, correlation matrix, no rotation) was used for miR-320a, miR-342, miR-146a, miR-378a, miR-375, miR-423a, miR-125b, miR-199 as an exploratory analysis to detect the underlying relationships among miRNAs and to identify cases clusters. Data assumptions were checked, KMO (Keiser Meyer Olkin) and Bartlett's test of sphericity were applied to test the suitability of the data for structure detection. Factor scores were calculated for dogs when the component's Eigen value was greater than one, to evaluate the distribution of the subjects according to the considered variables and classed using the categories healthy dogs and dogs with otitis.

To determine the diagnostic accuracy of targets differing statistically between healthy and otitis affected dogs, receiver operating characteristic (ROC) analysis was performed as previously reported (45). The diagnostic values were calculated for miRNAs that showed significant differential expression in the buffalo blood.

## Results

### Demographics and Characteristics of Study Subjects

A total of 95 samples, 59 from healthy and 36 from ears affected by otitis externa, were collected. The median age in the control and otitis affected groups was 9 (ranging from 6 months to 15 years) and 8 (ranging from 1 to 14 years) years, respectively. The male-to-female ratio was 11:17 in the healthy group and 11:10 in the otitis-affected group. A total of 19 different breeds was included in the list, with an over-representation of Labrador Retrievers (8) and German shepherds (5). The list of samples including the diagnosis and cytological and bacteriological data are listed in Supplementary Table 1.

### Cytology and Bacteriology

Cytological findings and yeast and bacterial isolation results are listed in Supplementary Table 1. Mites were not observed in any of the cytological specimens. Cytology and culture evidenced bacterial organisms (both coccoid and rod-shaped) and yeast in clinically healthy and otitis affected ears independently of the clinical presentation. As it could be assessed by cytology, bacteria were higher in diseased ears. Yeast numbers did not correlate with otitis. In three dogs with a clinical diagnosis of otitis, neutrophils phagocytizing bacteria were observed in high numbers and were consistent with the clinical finding of severe otitis. *Malassezia* and bacterial organisms were isolated from healthy and diseased ears and no association with otitis externa was observed between number and type of yeast or bacteria.

### RNA Extraction From Cerumen and Determination of miRNome Profile

To characterize miRNA expression profiles of cerumen, a pilot study small RNA-seq was performed on RNA extracted from the cerumen samples of three healthy and three otitis-affected dogs. After RNA extraction, small RNAs were selected according with their size (≈146 bp band) and sequenced on the NextSeq500 sequencer (Illumina). Multiple reads per sample, varying from 349,000 to 11,000,000, were obtained. Counts table was used to detect differentially expressed miRNAs via DESeq2 analysis (44). Furthermore, the analysis revealed the expression of 102 *Canis familiaris* (cfa) miRNAs, discarding lowly expressed miRNAs (≤1 raw count across 6 samples).

### MiRNAs Are Modulated in Cerumen of Otitis-Affected Dogs

**Figure 1:**
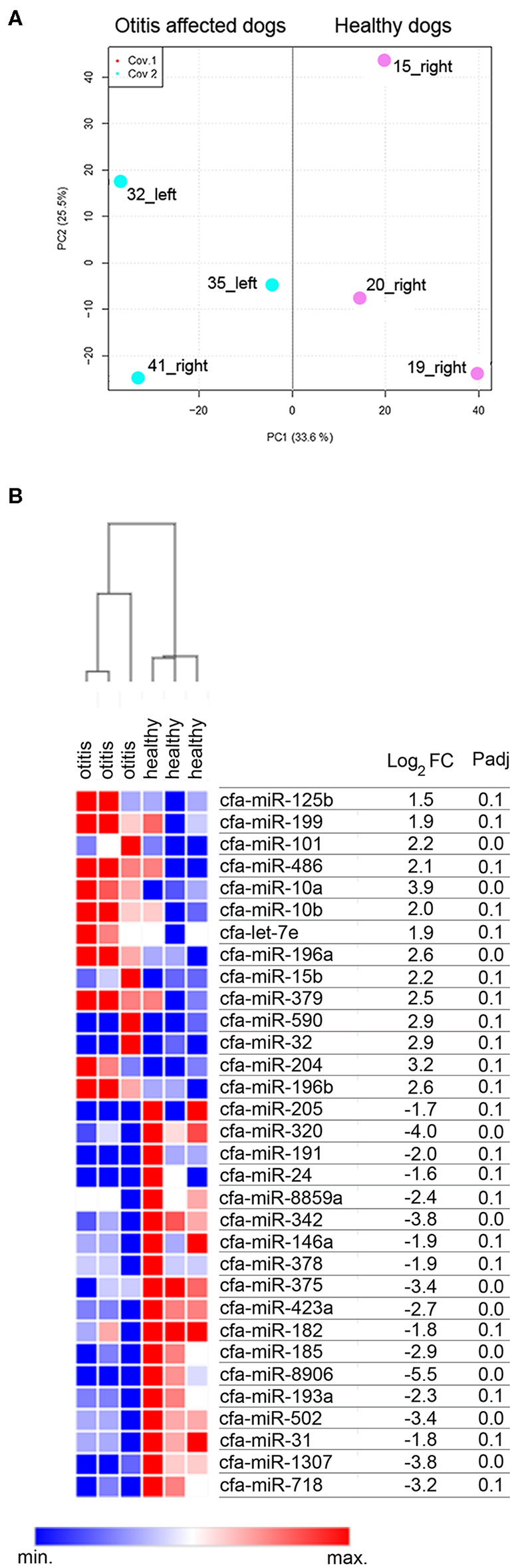
Cerumen sequencing results. **(A)** Principal Component Analysis (PCA) of six sequenced samples. Two-dimensional PCA was used to determine whether otitis affected (blue point) could be distinguished from healthy (pink points) subjects. **(B)** Identification of DE-miRNAs between otitis affected and healthy dogs. Heat-map and table displaying the fold change and Padj of DE-miRNAs.

A cluster analysis based on the expression profiles of the six sequenced samples was performed. The results allowed to differentiate the samples in two clusters, namely cluster of otitis-affected and cluster of healthy control group (Figure 1A). To determine whether there were differences in the miRNAs expression profile of healthy and otitis-affected samples, a differential expression (DE) analysis applying using DESeq2 (44), with a threshold adjusted *P* < 0.1 and |log_2_FC| >1, was performed. A difference in miRNA profiles was observed, suggesting molecular changes due to otitis externa. Thirty-two miRNAs were significantly altered in otitis-affected dogs, of which 14 resulted upregulated (1.5- to 3.9- fold) and 18 down-regulated (1.6- to 5.5- fold) (Figure 1B).

### Validation of Differentially Expressed miRNAs in Otitis-Affected and Healthy Dogs

**Figure 2:**
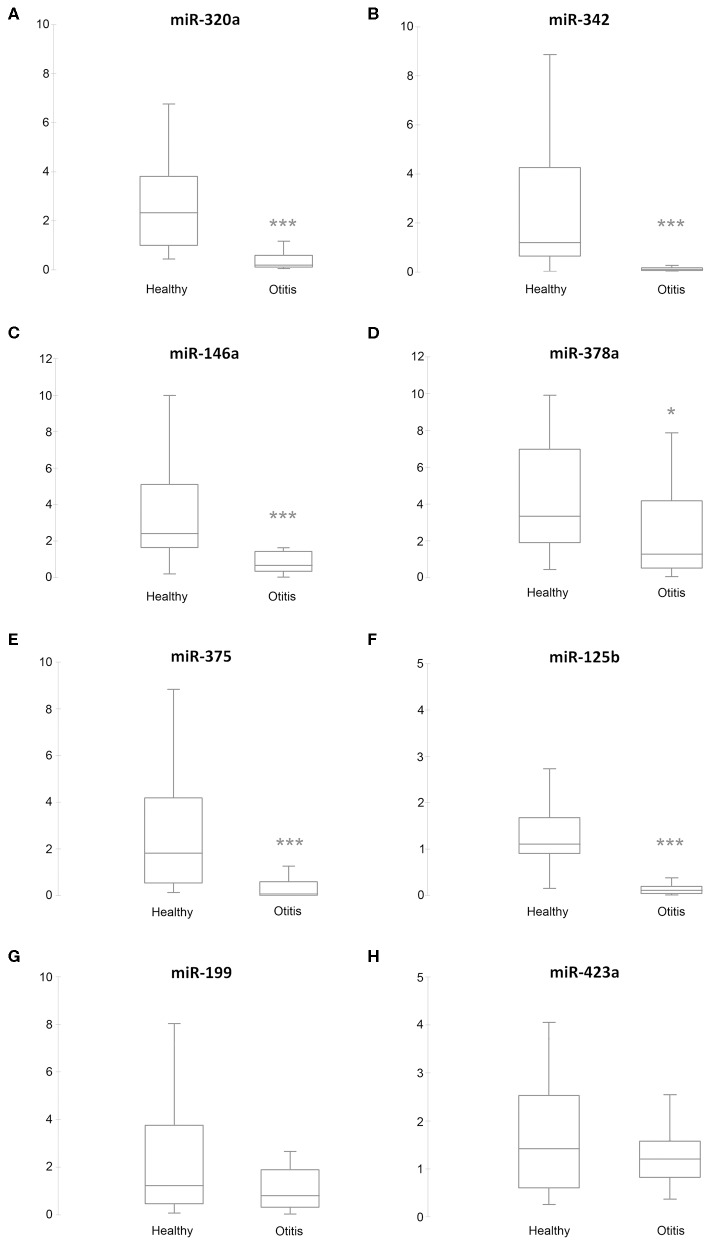
Box plots of DE-miRNAs in otitis affected compared with healthy dogs. Significance was declared at *P < 0.05, and ***P < 0.001. Black lines inside the boxes mark the medians. Whiskers indicate variability outside the upper and lower quartiles. **(A)** miR-320a, **(B)** miR-342, **(C)** miR-146a, **(D)** miR-378a, **(E)** miR-375, **(F)** miR-125b, **(G)** miR-199, and **(H)** miR-432a.

RT-qPCR validation was performed on the 6 sequenced samples and on a separate independent set of 89 samples, collected from 56 from healthy and 33 from otitis-affected ears. To validate the sequencing results, eight differentially expressed (DE)-miRNAs were selected following their potential involvement in regulating the immune system. Their relative abundance was quantified using RT-qPCR. MiR-21-5p, miR-26a-5p, and miR-27-3p were analyzed as endogenous controls for normalization. Cel-miR-39, an artificial spike-in, was used as an internal control. The results are presented in Figure 2. The selected miRNA targets were detected in all samples. In accordance with the sequencing data, RT-qPCR results demonstrated that the levels of five miRNAs (miR-320a: *P* ≤ 0.0001, ratio_healthy/Otitis_ = 17.5; miR-342: *P* ≤ 0.0001, ratio_healthy/Otitis_ = 15; miR-146a: *P* ≤ 0.0001, ratio_healthy/Otitis =_ 2.1; miR-378a: *P* = 0.0035, ratio_healthy/Otitis_ = 2.9; miR-375: *P* ≤ 0.0001, ratio_healthy/Otitis_ = 11) were significantly down-regulated in otitis-affected dogs. Remarkably, the RT-qPCR validation for miR-125b (*P* ≤ 0.0001, ratio_healthy/Otitis_ = 12.3) did not confirm the sequencing results, presenting the evidence that this miRNA is down-regulated. MiR-199 and miR-423a did not exhibit statistically significant differences between otitis affected and healthy dogs.

**Figure 3:**
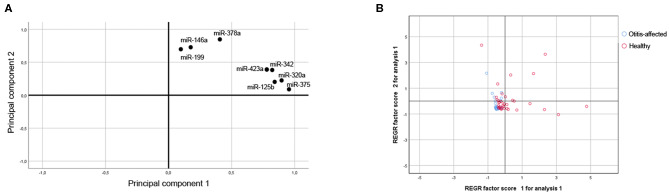
Multivariate statistical analysis. **(A)** Projection for the loadings of the miRNAs considered for the First and Second Principal Component. **(B)** Score plot of dogs in terms of clinical outcome.

The miR-320a, miR-342, miR-146a, miR-378a, miR-375, miR-423a, miR-125b, and miR-199 abundance for the 95 samples were also analyzed together using Principal Component Analysis (PCA, correlation matrix, no rotation), which is an exploratory analysis tool used to explain the structure of a set of variables through linear combinations. Good suitability of data for PCA analysis was valued (KMO = 0.795 and Bartlett's test *P* ≤ 0.001). The PCA revealed two main factors with Eigenvectors greater than one, which together explains 75.9% of the variation between dogs. As shown in Figure 3A, the first factor (PC1-Component 1; Eigenvalue = 3.927; Explained variance = 49.092%) shows positive loadings for miR-320a, miR-342, miR-375, miR-423a, and miR-125b. The second factor (PC2-Component 2; Eigenvalue = 2.143; Explained variance = 26.791%; Cumulative explained variance = 75.883%) shows positive loadings for miR-146a, miR-378a, and miR-199. To test whether there were any significant effects of the dog condition, the PC miRNAs scores attributed to the samples on the first two main components of the PCA (explaining 75.883% of total variance) were analyzed through a Kruskal-Wallis test. Based on the category healthy and otitis-affected, dogs did not cluster homogeneously but were significantly (*P* ≤ 0.001) sorted into two groups on PC1 (Figure 3B): one group with higher variable values associated with healthy dogs and the second group identified by a lower variability for otitis-affected dogs.

### Assessment of the Diagnostic Value of DE-miRNAs

**Figure 4:**
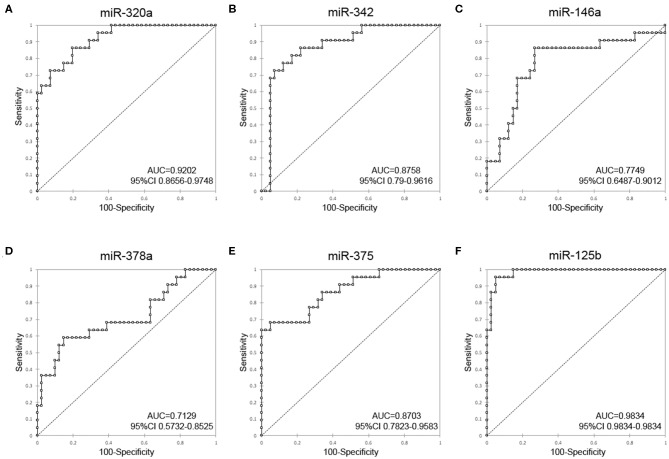
Receiver-operator characteristic (ROC) curve analysis of DE-miRNAs in the cerumen. AUC, area under the curve; CI, confidence interval. **(A)** miR-320a, **(B)** miR-342, **(C)** miR-146a, **(D)** miR-378a, **(E)** miR-375, and **(F)** miR-125b.

To investigate the diagnostic value and the diagnostic potency of DE-miRNAs in the cerumen, ROC curves and the area under the curve (AUC) were calculated. The diagnostic performance is reported in Table 1. The AUC was fair for miR-146a and miR-378a, good for miR-342 and miR-375, and excellent for miR-320a and miR-125b (Figure 4). Discriminant analysis was carried out to investigate the potential for improving diagnostic performance by analyzing multiple DE-miRNAs. The weighted average relative quantification (RQ) values of the miRNAs with an AUC>0.9 (miR-let-320a and miR-125b) and with AUC>0.8 (miR-let-320a, miR-125b, miR-342, and miR-375) were analyzed (Supplementary Figure 1). Median expression levels including the RQ of 2 DE-miRNAs were 26 (range, 17.07–703.13) and 3.2 (range, 0.74–17.74) in healthy and otitis affected dogs, respectively (Supplementary Figure 1A). Median expression levels including the RQ of 4 DE-miRNAs were 13.3 (range, 3.31–367.7) and 1.69 (range, 0.24–9.1) in healthy and otitis affected dogs, respectively (Supplementary Figure 1C). The predicted probability of being discriminated as infected from the logit model based on the two [logit = (19.5 × expression level of miR-320a) + (10.4 × expression level of miR-125a)] or the four cerumen DE-miRNAs [logit = (19.5 × expression level of miR-320a) + (10.4 × expression level of miR-125a) + (5.26 × expression level of miR-342) + (-4.55 × expression level of miR-375)] was used to construct the ROC curves (Supplementary Figures 1B,D). The results of ROC curves analysis are reported in Table 1.

**Table 1:** Area under the curve (AUC), sensitivity, specificity, and accuracy for DE-miRNAs in the cerumen.

**miRNA**	**AUC**	**95% CI**	***P*-value**	**Cut-off**	**Sensitivity**	**Specificity**	**Accuracy**
miR-320a	0.9202	0.8656–0.9748	<0.0001	0.9084	0.8636	0.9748	0.8254
miR-342	0.8758	0.79–0.9616	<0.0001	0.2308	0.7273	0.9268	0.8571
miR-146a	0.7749	0.6487–0.9012	<0.0001	1.7416	0.8636	0.7317	0.7778
miR-378a	0.7129	0.5732–0.8525	0.0028	1.5993	0.5909	0.8537	0.7619
miR-375	0.8703	0.7823–0.9583	<0.0001	0.1464	0.6364	1	0.8730
miR-125b	0.9834	0.9834–0.9834	<0.0001	0.4365	0.9545	0.9512	0.9524
Av_2	0.9607	0.9349–0.9865	<0.0001	10.28	0.8696	0.9286	0.9077
Av_4	0.9762	0.9762–0.9762	<0.0001	4.32	0.8696	0.9762	0.9385

*Av_2, weighted average relative quantification of miR-320a and miR-125b; Av_4, weighted average relative quantification of miR-320a, miR-125b, miR-342, and miR-375*.

### miRNA Localization, Target Prediction, and Pathway Enrichment

**Figure 5:**
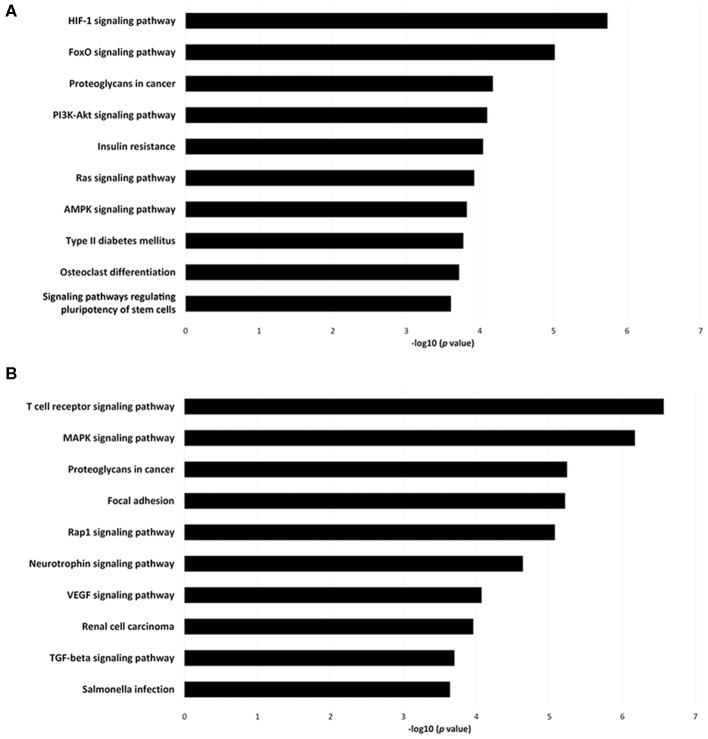
Pathway enrichment analysis for genes regulated by **(A)** up- and **(B)** down-regulated miRNAs. Genes regulated by DE-miRNAs were retrieved and enriched in KEGG using DAVID. The *P*-value was negative 10-base log transformed. The top 10 enriched KEGG pathways are reported.

**Figure 6:**
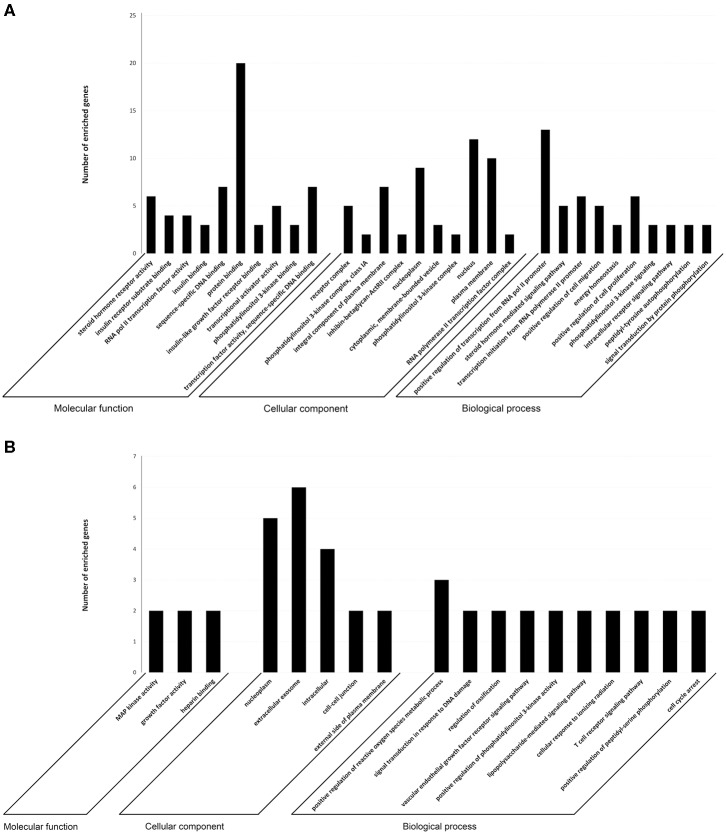
Target prediction. GO annotation of genes regulated by **(A)** up- and **(B)** down-regulated miRNAs. The target genes were annotated by DAVID at three levels: molecular function, cellular component, and biological process. The top 10 significantly enriched items are shown.

**Figure 7:**
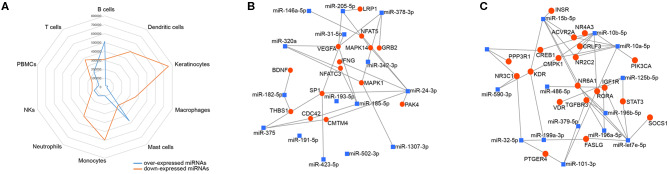
Localization of DE-miRNAs and network analysis of miRNAs-immune-related target genes in the cerumen. **(A)** FANTOM5 miRNAS atlas was analyzed for the expression of DE-miRNAs in immune cells and keratinocytes. Expression (counts per million) was plotted for these miRNAs in the radar graph; **(B)** network of over-expressed miRNAs constructed using miRNet; **(C)** network of down-regulated miRNAs constructed using miRNet. The blue squares represent miRNAs, the red dots mRNA.

To investigate the immune relevance, predicted targets of DE-miRNAs were computationally retrieved from miRWalk resources. The mRNA enrichment was performed using DAVID bioinformatic tool. Since little information on alterations in immune response contributing to the onset and progression of otitis externa are available, an enrichment of mRNA targets that encode for immunologically relevant genes was performed comparing the target genes obtained from miRWalk with the Gene List of ImmPort (46). The predicted mRNA targets of up-regulated miRNAs were 270 [164 at 3′ untranslated region (UTR), 21 at 5′UTR, and 85 at codon sequence (CDS)], of which 21 were immune-related. The predicted mRNA targets of down-regulated miRNAs were 133 (78 at 3′UTR, 10 at 5′UTR, and 45 at CDS), of which 15 were involved in immunity. The list of immunologically relevant genes is reported in Table 2. KEGG pathway analysis was performed on the enriched immune-related targets of up- and down-regulated miRNAs using DAVID. The top 10 significantly enriched KEGG pathways are reported in Figure 5. The up-regulated miRNAs (Figure 5A) were identified to be predominantly involved in the following pathways: HIF1 (Hypoxia Inducible Factor 1) and FoxO (Forkhead box O3) signaling pathways. The down-regulated miRNAs (Figure 5B) were revealed to be involved in the T cell receptor signaling pathway, MAPK (Mitogen-Activated Protein Kinase) signaling pathway, Focal adhesion, and RAP1 (Ras-proximate-1) signaling pathway. Aiming for further understanding the associated functions of the DE-miRNAs, Gene Ontology (GO) analysis was performed. GO enrichment analysis included the categories molecular function (MF), cellular component (CC), biological process (BP) (Figure 6). For down-regulated miRNAs, most MF items mainly included genes involved in the regulation of MAPK activity, growth factor activity, and heparin-binding; the enriched CC converged on genes associated with the nucleoplasm and extracellular exosomes, while BP on ROS (Reactive oxygen species) metabolic species, signal transduction in response to DNA damage, and VEGF (Vascular-Endothelial Growth Factor) receptor signaling pathway. For up-regulated miRNAs, MF items focused on steroid hormone receptor activity and insulin receptor substrate binding; CC converged on receptor complex, phosphatidylinositol 3-kinase complex and integral component of plasma membrane, and BP on positive regulation of transcription from RNA polymerase II promoter, steroid hormone-mediated signaling pathway, and positive regulation of cell migration. To identify which type of cells in cerumen express DE-miRNAs, the atlas of miRNA expression (FANTOM5) in immune cells and keratinocytes was explored. The radar chart reported in Figure 7A presents the contribution of cells to the production of up- and down-regulated miRNAs. In detail, down-regulated miRNAs are produced mainly by keratinocytes, monocytes, dendritic cells, and T cells, while up-regulated miRNAs by B cells and mast cells. The miRNA–mRNA networks determined using miRNet database are presented in Figures 7B,C.

**Table 2:** Immune-related target genes of differentially expressed miRNAs.

**Immune-related genes targeted by up-regulated miRNAs**	**Immune-related genes targeted by down-regulated miRNAs**
*FASLG, IGF1R, TGFBR3, SOCS1, CRLF3, NR2C2, RORA, NR4A3, CREB1, STAT3, VDR, INSR, ACVR2A, KDR, NR3C1, PTGER4, EDN1, PIK3R1, NR6A1, PIK3CA, PPP3R1*	*BDNF, THBS1, CDC42, VEGFA, IFNG, PDGFRB, MAPK14, PAK4, CMTM4, GRB2, LRP1, NFATC3, NFAT5, SP1, MAPK1*

## Discussion

The findings of this study provided for the first-time evidence that (a) miRNAs can be efficiently extracted, sequenced, and quantified by RT-qPCR from canine cerumen and (b) cerumen microRNAs quantities change during otitis externa. In the first part of the investigation, a pilot sequencing study was performed to profile the miRNome of cerumen, showing that otitis externa changed the expression of 32 miRNAs, of which 14 were more abundant and 18 were less abundant compared to healthy dogs. In the second step, 8 differentially expressed miRNAs were validated on a larger cohort using a RT-qPCR approach. It was found that miR-320a, miR-342, miR-146a, miR-378a, miR-375, and miR-125b were down-regulated in cerumen from otitis affected dogs. The results are supported by PCA analysis, of which the first principal component accounts for as much of the variability in the data as possible. miRNAs on this component are the most important in explaining the differences between healthy and otitis-affected dogs. Moreover, heat map, hierarchical clustering, and PCA revealed that dogs with otitis showed increased variability in miRNA levels compared to healthy ones.

Given the functions of the target genes regulated by DE-miRNAs, the current findings demonstrated that miRNAs contained in cerumen might interact with several pathways involved in the host innate immunity, including modulation the inflammatory reaction, the regulation of M1/M2 monocyte lineage polarization, the resolution of inflammation, and reparation of damaged tissues.

All DE-miRNAs are involved in pathways that regulate the inflammatory reaction. Therefore, their down-regulation provides cerumen with a potential pro-inflammatory activity, following a mechanism different for each DE-miRNAs. For example, the downregulation of miR-320a induced the overexpression of pro-inflammatory cytokines through promoting *COX-2* (*Cyclooxygenase-2*) expression by targeting *MAPK-1* (47). In macrophages, decreasing miR-125b-5p has a dual, apparently opposite, effect of increasing secretion of the pro-inflammatory chemokine *MCP-1* (*Monocyte chemoattractant protein-1*) (48) and upregulating *B7-H4* in macrophages, which induces an anti-inflammatory effect (49). Decreasing miR-378a through targeting CD47-*SIRP*α (Signal Regulatory Protein Alpha) inhibits phagocytosis in macrophages, and promotes the secretion of *TNF*α (*Tumor Necrosis Factor-alpha*) and *IL-6* (*Interleukin-6*) (50). MiR-375 regulates the expression of pro-inflammatory cytokines such as *IL1-*β*, TNF*α, and *IL-6*: therefore, miR-375 decrease also reduces cytokine expression, as shown in a myocardial infarction model (51). Finally, miR-146 is involved in the regulation of inflammation via negative feedback of toll-like receptor signaling (*TLR*) (52), as already described in otitis media in humans (24): consequently, down-regulation of miR-146 induces a pro-inflammatory effect. Moreover, miR-125b and miR-146, which are also reduced after TLR activation, can promote tolerance to endotoxin (53). We found that all these miRNAs are less abundant in cerumen of dogs affected by otitis, confirming what has been already reported in other diseases such as for miR-342 (54) and miR-375 in sepsis, or during C5a (complement component C5a) activation for miR-320a (55).

All DE-miRNAs are also involved in the modulation of monocyte/macrophage polarization. Specifically, miR-125b, miR-378, miR-375, and miR-372 are involved in polarization to M1 lineage whereas miR-320a and miR-146a are involved in polarization toward M2 lineage. MiR-320a, in particular, was found in epithelial-derived microvesicles, and could activate macrophage pro-inflammatory effects (56). The effect of miR-320a may be even more complex since a more recent study demonstrated that miR-320a promotes the polarization toward immunosuppressive M2 macrophages meanwhile inducing polarization toward M1 lineage (57). Moreover, the effects of miR-375 require a more in-depth investigation since the inhibition of miR-375 represses M1 macrophage polarization and promotes M2 macrophage polarization, targeting *PDK-1* (*Pyruvate Dehydrogenase Kinase 1*) (51).

Following their inflammatory functions, classical M1 macrophages feature higher capabilities of phagocytosis and, more in general, a pro-inflammatory phenotype. On the contrary, non-classical M2 monocytes share a lower pro-inflammatory activity, although their precise physiological roles remain still poorly defined (58). Given the background that miRNAs play pivotal roles in macrophage activation and polarization (59), the finding that miRNAs involved in monocyte/macrophage polarization were detected in cerumen was not surprising and suggests that during inflammatory responses, monocytes are attracted to cerumen, and become activated on site and modulated by miRNAs.

The DE-miRNAs are involved in a third mechanism represented by the regulation of repair pathways after inflammation. For example, miR-320a is involved in intestinal mucosal reconstitution and repair after inflammation (60), and miR-125b inhibits proliferation and promotes differentiation of keratinocytes in the skin (61). Therefore, the capability of cerumen to down-regulate miR-320a and miR-125b may result in keratinocyte proliferation, which in turn may accelerate wound healing and homeostasis restitution.

Although neutrophils were observed in high numbers only in three dogs, cytological findings associated always with otitis paralleling the observations of Angus (62). According to our results, cytology should be considered a specific diagnostic technique assisting in the diagnosis of otitis in dogs although bearing lower sensitivity. Isolation of yeasts and bacteria species did not correspond to a specific condition; however, increased numbers of bacteria were evidenced in cases of otitis as previously reported (62). These findings suggested that isolation of organisms should be assessed in the context of clinical presentation and cytological findings as an adjunctive tool to support diagnostic and therapeutic protocols. Noteworthy, cytology and microbiology did not always result in sensitive techniques to distinguish healthy vs. diseased ears. On the contrary, ROC analysis highlighted that two miRNAs, namely miR-125b and miR-320a, can discriminate otitis-affected from healthy dogs with high sensitivity (>86%) and specificity (>97%), confirming that these miRNAs may be excellent candidate biomarkers. These findings are more relevant if considered that differentiation among diseased and normal ears occurred independently from the presence of elevated numbers of neutrophils thus independently of morphological features of inflammation.

Currently, one of the main issues in human as well as in veterinary medicine is the overuse of antibiotics, which promotes the selection of resistant commensal flora. Careful use of antibiotics for treatment of cutaneous infections, including otitis externa in dogs, is recommended (63). We believe that molecular biomarkers, such as the miRNAs identified in this work, may assist the clinical monitoring of drug effectiveness during otitis externa treatment. To support this hypothesis, further studies will be performed on cerumen collected from dogs affected by otitis and treated with antibiotics. Moreover, as allergic dermatitis is the most frequently recognized primary cause of canine otitis externa (64), further studies evaluating the change of biomarkers expression in allergic dogs, without symptomatic otitis externa, compared to healthy subjects could provide the clinician with a valuable screening method to monitor the ear canal inflammatory status. This could, in turn, support the proactive use of targeted anti-inflammatory treatments aimed to prevent the development of secondary infections decreasing further the use of antibiotics and the risk of bacterial resistance.

In conclusion, to the best of the authors' knowledge, this is the first report demonstrating the presence of miRNAs in cerumen and their changes in dogs with acute otitis externa. These findings provided insights on the role of miRNAs in modulating immune defenses in cerumen, a biological fluid whose importance has been almost completely neglected so far, meanwhile highlighting the potential role of cerumen as a source of biomarkers. In this work, the finding of miRNAs differential expression is relevant for a better understanding of the pathogenic mechanism leading to otitis and tampering of external ear damage and provides a novel technique able to discriminate healthy vs. otitis-affected ears representing a more specific and sensitive diagnostic tool compared to cytology and microbiology. Thus, the abnormal expression of miRNAs may lead to an early diagnosis of otitis and timely treatment.

Further studies are necessary to confirm their diagnostic values by increasing the number of clinical samples, associating their abundance with specific pathogens and antibiotic treatment, and to investigate the direct interaction between these miRNAs and their target genes.

## Data Availability Statement

The raw data supporting the conclusions of this article will be made available by the authors, without undue reservation, to any qualified researcher.

## Ethics Statement

All applicable international, national, and/or institutional guidelines for the care and use of animals were followed. Samples were collected during routine health checks or clinical evaluation of affected dogs, under informed consent of the owners and out of the scope of Directive 2010/63/EU (art. 1.5.f practices not likely to cause pain, suffering, distress or lasting harm equivalent to, or higher than, that caused by the introduction of a needle in accordance with good veterinary practice). This study design was approved by the Italian Ministry of Health, project n. IZS ME 13_15 RC.

## Author Contributions

CL and FC conceived and designed the experiments and provided the original idea of the study. PR, RP, SL, and ED'U enrolled patients and performed the clinical diagnosis. MC and GGr carried out the cytology and microbiological analysis, respectively. MA performed the sequencing experiment. RC, MM, and CL performed bioinformatics and statistical analysis. CL and VZ performed the RT-qPCR experiments. FC, GGa, and GB funded the project. CL wrote the first draft of the manuscript. All authors read and approved the final draft of the manuscript.

## Conflict of Interest

The authors declare that the research was conducted in the absence of any commercial or financial relationships that could be construed as a potential conflict of interest.
