# Supplementary material for: Identification of Altered miRNAs in Cerumen of Dogs Affected by Otitis Externa
Source: Front Immunol. 2020 May 29;11:914. doi: 10.3389/fimmu.2020.00914 (PMC7273745; doi:10.3389/fimmu.2020.00914)
Supplement: Supplementary file 1 [file Table_1.pdf]

Supplementary table 1. Signalment and clinical data of dogs enrolled in the study.

	Breed	Gender	Age (Years)	Ear	Clinical diagnosis	Cellularity	Epithelium	Immune cells	Malassezia spp	Bacteria	Bacterial culture results
**1**	Weimaraner	male	9	Right	Healthy	3	3	0	3	1	Staph. pseudintermedius
				Left	Healthy	4	4	0	3	1	Staph. pseudintermedius
**2**	Mongrel	female	11	Right	Healthy	3	3	0	3	1	Staph. xylosus
				Left	Healthy	4	4	0	3	1	Proteus mirabilis
**3**	Jack russell terrier	female	13	Right	Healthy	2	2	0	0	1	Staph. spp
				Left	Healthy	3	3	0	0	1	Staph. spp
**4**	Maltese	male	2	Right	Healthy	2	2	0	0	1	Staph. xylosus
				Left	Healthy	2	2	0	0	1	Staph. xylosus
**5**	Mongrel	male	3.5	Right	Healthy	2	2	2	0	1	Strep. mitis
				Left	Healthy	2	2	0	0	1	Strep. spp
**6**	Not reported	female	10	Right	Healthy	2	2	0	0	1	Staph. pseudintermedius
				Left	Healthy	2	2	0	0	1	Unidentified cocci
**7**	German shepherd	female	9	Right	Healthy	4	4	0	4	2	Staph. pseudintermedius
				Left	Healthy	4	4	0	4	2	Staph. pseudintermedius
**8**	Labrador Hovawart	male	8	Right	Healthy	2	2	0	0	1	Unidentified cocci
				Left	Healthy	2	2	0	1	1	Strep. canis
**9**	Beagle	male	10	Right	Healthy	2	2	0	1	1	Strep. mitis
				Left	Healthy	2	2	0	1	1	Strep. mitis
**10**	German shepherd	female	11	Right	Healthy	2	2	0	0	4	Staph. pseudintermedius
				Left	Healthy	2	2	0	1	1	Unidentified cocci
**11**	Boxer	female	7	Right	Healthy	2	2	0	3	1	Staph. pseudintermedius
				Left	Healthy	2	2	0	3	1	Unidentified cocci
**12**	Not reported	male	1.5	Right	Healthy	4	4	0	0	0	Negative
				Left	Healthy	3	3	0	1	3	Staph. pseudintermedius
**13**	Cocker	male	15	Right	Healthy	4	4	0	4	1	Citrobacter spp.
				Left	Healthy	4	4	0	0	3	Pseudomonas aeruginosa

**14**	Not reported	female	0.5	Right	Healthy	2	2	0	0	3	Pseudomonas aeruginosa
				Left	Healthy	2	2	0	2	2	Staph. pseudintermedius
**15**	Labrador retriever	female	8	Right	Healthy	1	1	0	0	1	Negative
				Left	Healthy	1	1	0	0	1	Negative
**16**	Bouvier des Flandres	female	11	Right	Healthy	4	4	0	4	4	Staph. pseudintermedius
				Left	Healthy	4	4	0	4	2	Staph. pseudintermedius
**17**	Not reported	male	9	Right	Healthy	4	4	0	4	2	Proteus mirabilis
				Left	Healthy	4	4	0	0	2	Staph. xylosus
**18**	Golden retriever	female	10	Right	Healthy	3	3	0	4	2	Staph. pseudintermedius
				Left	Healthy	3	3	0	4	2	Staph. pseudintermedius
**19**	Golden retriever	male	6	Right	Healthy	3	3	0	0	1	Unidentified cocci
				Left	Healthy	3	3	0	1	1	Strep. spp
**20**	Mongrel	female	7	Right	Healthy	3	3	0	1	1	Staph. spp
				Left	Healthy	4	4	0	3	1	Enterococcus spp.
**21**	Brittany Spaniels	female	10	Right	Healthy	3	3	0	0	1	Proteus mirabilis
				Left	Healthy	2	2	0	0	1	Proteus mirabilis
**22**	Flat coated retriever	male	1	Right	Healthy	3	3	0	4	1	Staph. pseudintermedius
				Left	Healthy	3	3	0	4	1	Staph. pseudintermedius
**23**	Labrador	female	1	Right	Healthy	4	4	0	1	1	Micrococcus *spp*.
				Left	Healthy	4	4	0	0	1	Staph. hyicus
**24**	Pitbull	female	1	Right	Healthy	4	4	0	0	1	Staph. pseudintermedius
				Left	Healthy	4	4	0	3	1	Pseudomonas fluorescens
**25**	French bulldog	Not reported	1.5	Right	Healthy	4	4	1	0	2	Staph. pseudintermedius
				Left	Healthy	4	4	0	0	1	Enterococcus faecalis
**26**	Mongrel	female	11	Right	Healthy	4	4	0	2	1	Enterococcus faecalis
				Left	Not collected						
**27**	Golden retriever	male	10	Right	Healthy	3	3	0	0	1	Staph. pseudintermedius
				Left	Healthy	4	4	0	1	1	Staph. pseudintermedius
**28**	Not reported	female	12	Right	Healthy	4	4	0	0	1	Strep. *spp*
				Left	Healthy	4	4	0	0	1	Strep. *spp*
**29**	Rhodesian Ridgeback	male	1.5	Right	Otitis	4	4	0	4	1	Staph. *spp*

				Left	Healthy	4	4	0	4	1	Staph. *spp*
**30**	Cocker	male	14	Right	Otitis	4	4	0	4	0	Staph. *spp*
				Left	Otitis	4	4	0	0	1	Micrococcus *spp.*
**31**	Not reported	male	6	Right	Otitis	2	2	0	1	1	Enterococcus faecalis
				Left	Otitis	2	2	0	1	1	Enterococcus spp.
**32**	Dalmatian	female	8	Right	Otitis	4	4	0	2	2	Staph. pseudintermedius
				Left	Otitis	4	4	0	4	2	Staph. pseudintermedius
**33**	Labrador	male	9	Right	Healthy	2	2	0	1	1	Staph. pseudintermedius
				Left	Otitis	4	4	2	4	4	Staph. pseudintermedius
**34**	Mongrel	female	10	Right	Otitis	4	3	0	4	3	Staph. pseudintermedius
				Left	Healthy	4	4	0	0	1	Micrococcus *spp*
**35**	Mongrel	female	8,5	Right	Otitis	4	4	0	4	1	Strep. *spp*
				Left	Otitis	4	4	0	4	1	E. coli
**36**	French bulldog	female	1	Right	Otitis	3	3	0	3	3	Staph. pseudintermedius
				Left	Otitis	3	3	0	4	3	Staph. pseudintermedius
**37**	Labrador	male	2	Right	Otitis	3	3	0	4	1	Staph. aureus
				Left	Otitis	3	3	0	2	3	E. coli
**38**	German shepherd	female	3	Right	Otitis	3	3	0	4	2	Proteus *spp*
				Left	Otitis	4	1	4	2	4	Pseudomonas aeruginosa
**39**	Mongrel	male	8	Right	Healthy	1	1	0	0	1	Citrobacter *spp*
				Left	Otitis	1	1	0	0	0	Negative
**40**	West highland White Terrier	Not reported	7	Right	Otitis	1	1	0	0	4	Staph. pseudintermedius
				Left	Otitis	1	1	0	0	4	Staph. pseudintermedius
**41**	German shepherd	female	11	Right	Otitis	0	0	0	0	0	Negative
				Left	Otitis	4	4	0	0	1	Negative
**42**	Labrador		9	Right	Otitis	4	4	0	1	4	Staph. pseudintermedius
				Left	Otitis	4	4	0	1	4	Staph. pseudintermedius
**43**	Cocker	male	8	Right	Otitis	3	3	0	1	3	Pseudomonas aeruginosa
				Left	Otitis	3	3	0	1	3	Staph. aureus
**44**	German shepherd	female	9	Right	Otitis	3	3	0	1	4	Staph. pseudintermedius
				Left	Otitis	3	3	0	1	4	Staph. pseudintermedius

**45**	English bulldog	male	11	Right	Otitis	3	3	0	0	4	Staph. aureus
				Left	Otitis	3	3	0	0	4	Pseudomonas aeruginosa
**46**	Labrador retriever	female	1	Right	Otitis	4	4	0	3	2	Staph. pseudintermedius
				Left	Otitis	4	4	0	4	0	Negative
**47**	Labrador	male	2	Right	Otitis	4	4	4	0	4	Staph. aureus
				Left	Otitis	3	3	0	3	1	E. coli
**48**	West Highland White Terrier	male	7	Right	Otitis	4	3	3	0	4	Staph. aureus
				Left	Otitis	3	3	0	4	3	Staph. pseudintermedius
