# Supplementary material for: Identification of Altered miRNAs in Cerumen of Dogs Affected by Otitis Externa
Source: Front Immunol. 2020 May 29;11:914. doi: 10.3389/fimmu.2020.00914 (PMC7273745; doi:10.3389/fimmu.2020.00914)
Supplement: Supplementary file 2 [file Image_1.pdf]

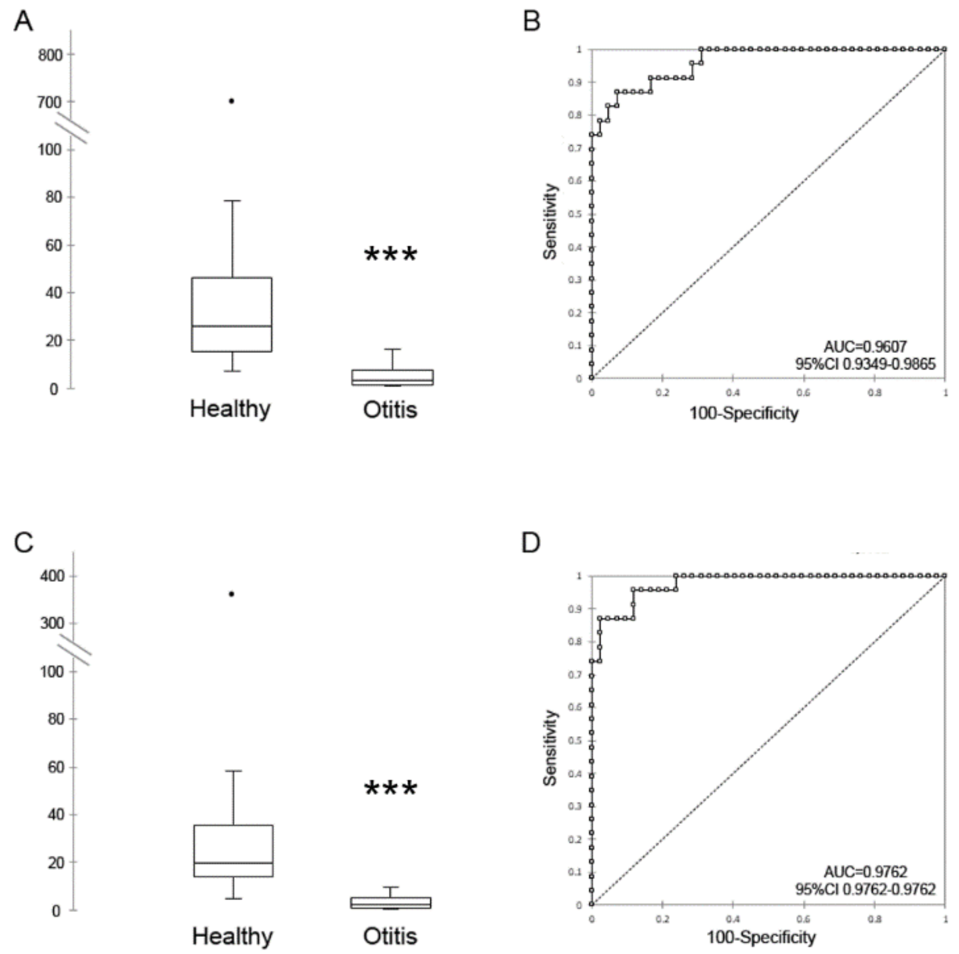

Supplementary figure 1. Average expression of the DE-miRNAs in the cerumen. Weighted average relative quantification (RQ) values of two- miR-320a and miR-125b- (A) and four - miR-320a, miR-125b, miR-375 and miR-342- (C) DE-miRNAs. ROC curve analysis, constructed using the logit model, for two - miR-320a and miR-125b- (B) and four - miR-320a, miR-125b, miR-375 and miR-342- (D) DE-miRNAs. AUC, area under the curve; CI, confidence interval. Black lines mark the medians. *** $P< 0.0001$.
